# Phylogenetic Analysis and Molecular Dating Suggest That *Hemidactylus anamallensis* Is Not a Member of the *Hemidactylus* Radiation and Has an Ancient Late Cretaceous Origin

**DOI:** 10.1371/journal.pone.0060615

**Published:** 2013-05-16

**Authors:** Rohini Bansal, K. Praveen Karanth

**Affiliations:** Centre for Ecological Sciences, India Institute of Science, Bangalore, India; George Washington University, United States of America

## Abstract

**Background of the Work:**

The phylogenetic position and evolution of *Hemidactylus anamallensis* (family Gekkonidae) has been much debated in recent times. In the past it has been variously assigned to genus *Hoplodactylus* (Diplodactylidae) as well as a monotypic genus ‘*Dravidogecko*’ (Gekkonidae). Since 1995, this species has been assigned to *Hemidactylus*, but there is much disagreement between authors regarding its phylogenetic position within this genus. In a recent molecular study *H. anamallensis* was sister to *Hemidactylus* but appeared distinct from it in both mitochondrial and nuclear markers. However, this study did not include genera closely allied to *Hemidactylus*, thus a robust evaluation of this hypothesis was not undertaken.

**Methods:**

The objective of this study was to investigate the phylogenetic position of *H. anamallensis* within the gekkonid radiation. To this end, several nuclear and mitochondrial markers were sequenced from *H. anamallensis*, selected members of the *Hemidactylus* radiation and genera closely allied to *Hemidactylus*. These sequences in conjunction with published sequences were subjected to multiple phylogenetic analyses. Furthermore the nuclear dataset was also subjected to molecular dating analysis to ascertain the divergence between *H. anamallensis* and related genera.

**Results and Conclusion:**

Results showed that *H. anamallensis* lineage was indeed sister to *Hemidactylus* group but was separated from the rest of the *Hemidactylus* by a long branch. The divergence estimates supported a scenario wherein *H. anamallensis* dispersed across a marine barrier to the drifting peninsular Indian plate in the late Cretaceous whereas *Hemidactylus* arrived on the peninsular India after the Indian plate collided with the Eurasian plate. Based on these molecular evidence and biogeographical scenario we suggest that the genus *Dravidogecko* should be resurrected.

## Introduction

*Hemidactylus anamallensis*, a gekkonid endemic to the Western Ghats of South India has undergone many taxonomic revisions, yet its phylogenetic position and taxonomic status remains unresolved. This species was originally described as a member of *Hoplodactylus* [1], [2], a genus in the family Diplodactylidae that is confined to New Zealand. Smith [3] assigned it to a new monotypic genus ‘*Dravidogecko*’ on the basis of the differences in subdigital pads and the arrangement of preanal pores, in the family Gekkonidae. Underwood [4] and Kluge [5] also demonstrated that *Dravidogecko* was a gekkonid gecko and not a member of the family Diplodactylidae. Russell [6], [7] on the basis of digital structure hypothesised that *Dravidogecko* was closely related to *Hemidactylus* group within family Gekkonidae. Later, Bauer and Russell [8] synonymised *Dravidogecko* as *Hemidactylus*, renaming it as *Hemidactylus anamallensis*, because there were no morphological features that were unique to *Dravidogecko* when compared with *Hemidactylus*. They also suggested that *H*. *anamallensis* could be a primitive *Hemidactylus*.

*Hemidactylus* is a species rich genus with 122 recognised species [9] distributed worldwide and has been identified predominantly on the basis of its phalangeal taxonomy [3], [6], [10], [11]. Russell [6] suggested that the genera *Briba*, *Cosymbotus*, *Dravidogecko* and *Teratolepis* also belong to *Hemidactylus*. Carranza and Arnold [12] undertook one of the most comprehensive phylogenetic studies of *Hemidactylus* based on mitochondrial 12S rRNA and cytochrome *b* sequenced from 30 species sampled from around the world. Their phylogeny retrieved five well supported clades. Three subsequent studies that included additional species (around 14) also retrieved the aforementioned clades [13]–[15]. In Carranza and Arnold [12] phylogeny *Cosymbotus* (distributed in Southeast Asia) and *Briba* (monotypic genus from Brazil) were deeply nested within the *Hemidactylus* group, hence they synonymised these genera with *Hemidactylus*. Bauer et al. [13], using molecular data from five genes, showed that *Teratolepis* was deeply embedded within the tropical Asian clade of *Hemidactylus* along with the ground dwelling geckos endemic to Indian subcontinent. Therefore, they synonymised it with *Hemidactylus*, renaming it as *Hemidactylus imbricatus*. These studies did not include *H*. *anamallensis*. Thus, its affinity to *Hemidactylus* based on morphological data needs to be evaluated using molecular data.

Within the *Hemidactylus* radiation, *H. anamallensis* has been assigned to the *H. bowringii* complex in the tropical Asian clade by Zug et al. [16]. Whereas Bauer et al. [13] suspected that *H. anamallensis* is part of a highly derived lineage, consisting of *H. albofaciatus-imbricatus-reticulatus* within the *H. brookii* complex in the tropical Asian clade. Thus, both the above scenarios would predict *H. anamallensis* to be deeply nested within the *Hemidactylus* radiation, but differ with respect to its exact phylogenetic position. These scenarios are in sharp contrast to Bauer and Russell's [8] hypothesis, wherein they considered *H. anamallensis* to be a primitive *Hemidactylus*, thereby suggesting that phylogenetically it could be sister to all the *Hemidactylus* species. These putative phylogenetic positions of *H. anamallensis* generate very different biogeographical scenarios for the origin and spread of both *H. anamallensis* and other *Hemidactylus* species of the Indian subcontinent. Interestingly, in a recent molecular work by Bansal and Karanth [15], *H. anamallensis* was indeed sister to all the *Hemidactylus* thus supporting Bauer and Russell [8] hypothesis. Nevertheless their results also suggested that “*H. anamallensis*” was genetically distinct from other *Hemidactylus*. However, in their study genera closely allied to *Hemidactylus* were not included, thus a robust evaluation of the phylogenetic position of *H. anamallensis* with respect to the genus *Hemidactylus* could not be undertaken. Therefore, the authors called for a re-examination of its allocation to the genus *Hemidactylus* with additional molecular data from related genera.

The objective of this study was to investigate the phylogenetic position of *H. anamallensis* within the gekkonid radiation. To this end, several nuclear and mitochondrial markers were sequenced from multiple *H. anamallensis* samples and these sequences were combined with published sequences of gekkonids. These alignments were then subjected to multiple phylogenetic analyses. Results from these analyses in conjunction with molecular dating were used to understand the origin and biogeography of *H. anamallensis*.

## Results

### Phylogenetic position of *H. anamallensis* within Gekkonidae (*C-mos* and *12S rRNA* dataset)

**Figure 1:**
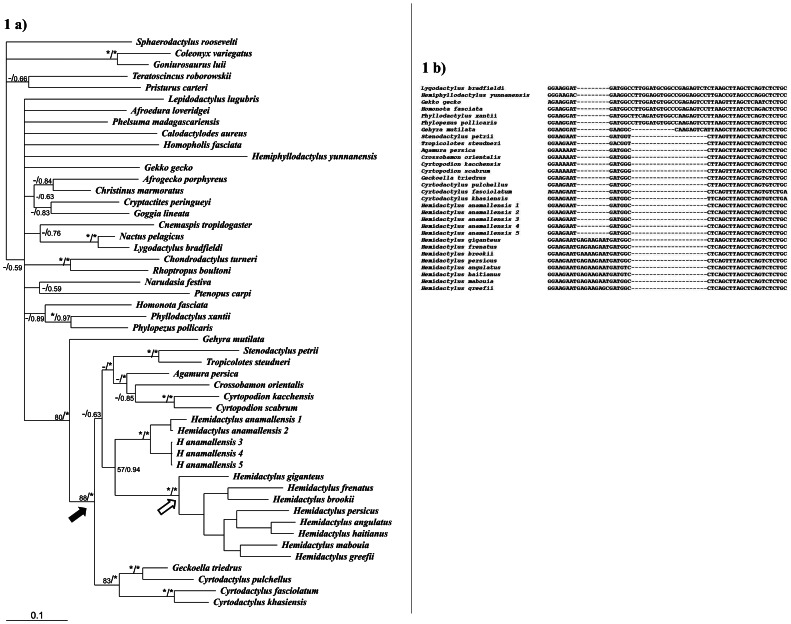
(a): Bayesian tree based on combined dataset of *C-mos* and *12S rRNA* genes showing the relationships among the members of the family Gekkoninae. The numbers at the nodes represent the maximum likelihood bootstrap/posterior probability. */* Indicates the bootstrap support ≥90%/Bayesian posterior probability of 1, -/indicates bootstrap support ≤50% and Bayesian posterior probability of <0.5. Black arrow represents the node that constitutes the members of the deletion clade and the white arrow represents the node, which separates the taxa with insertion (*Hemidactylus*). (b) *C-mos* DNA sequence alignment-showing indels among some members of the family Gekkoninae.

All tree building methods retrieved a strongly supported clade consisting of the genera *Agamura, Crossobamon, Cyrtodactylus, Cyrtopodian, Geckoella, Hemidactylus, Stenodactylus* and *Tropicolotes*. Members of this clade, henceforth referred to as deletion clade, also shared a 21 bp deletion in the *C-mos* gene (Bayesian tree shown in figure 1a and b). The relationships between members of the deletion clade were also identical across tree-building methods. Within the deletion clade, *Hemidactylus* (excluding *H. anamallensis*) formed a clade with high support. Additionally it was observed that the members of this *Hemidactylus* clade shared a unique 9 bp insertion in the *C-mos* gene (figure 1b). However, this insertion was not seen in *H. anamallensis*. In all the trees *H. anamallensis* emerged as sister to the rest of the *Hemidactylus* radiation. For a list of sequences used and their accession numbers see table 1.

**Table 1:** List of sequences used in the current study.

	GenBank Accession numbers					
Sample name	Voucher Number	Locality	C-mos	12S rRNA	RAG-1	PDC
*Aristelliger lar*	JB 01	Dominican Republic	-	-	EF534805	EF534847
*Bavayia cyclura*	AMB 7683	New Caledonia	-	-	HQ 426264	HQ 426176
*Blaesodactylus antongilensis*	ZCMV 2187	Nosy Mangabe, Madagascar	-	-	EU054229	EU054205
*Blaesodactylus sakalava*	WRBM 18	Will's Track, Toliara District, Madagascar	-	-	EU054227	EU054203
*Carphodactylus laevis*	AMS 143258	Queensland, Australia	-	-	EF534781	EF534821
*Cnemaspis limi*	LLG 6267	Pulau Tioman, Malaysia	-	-	EF534809	EF534851
*Coleonyx variegatus*	CAS 205334	California, USA	-	-	EF 534777	EF 534817
*Cyrtodactylus ayeyawardyensis*	CAS 216446	vic. Kanthaya Beach, Rakhine State, Myanmar	-	-	EU268287	EU268317
*Cyrtodactylus consobrinus*	LLG 4062	Niah Cave, Sarawak, Malaysia	-	-	EU268288	EU268318
*Cyrtodactylus fasciotlatus*	CES 091196	Kempty road, Mussoorie, Uttarakhand, India	KC735108	KC735096	HM622351	HM622366
*Cyrtodactylus gubernatoris*	CES 1197	Singhtum, Sikkim	-	-	KC735086	KC735091
*Cyrtodactylus khasiensis*	CES 1101	Northeast India	KC735109	KC735097	-	-
*Cyrtodactylus loriae*	FK 7709	N slope of Mt. Simpson, Bunisi, Milne Bay Province, Papua New Guinea	-	-	EU268289	EU268319
*Cyrtopdian scrabum*	CES1104	Sam, Rajasthan	KC735110	KC735098	-	-
*Cyrtopodian kacchensis*	CES1146	Kutch, Gujarat	KC735111	KC735099	-	-
*Cyrtopodian species*	CES1107	Kuno, Madhya Pradesh	KC735112	KC735100	-	-
*Delma Tincta*	AMS 151607	Sturt Natl. Pk., NSW, Australia	-	-	HQ 426277	HQ 426188
*Diplodactylus conspicillatus*	AMS 158426	Sturt Natl. Park, NSW, Australia	-	-	HQ 426278	HQ 426189
*Elgaria kingii*	TG 00065	Navajo County, Arizona, USA	-	-	AY662603	HQ426252
*Eublepharis macularius*	JS 2	Pakistan	-	-	EF 534776	EF 534816
*Euleptes europea*	-	Liguria, Italy	-	-	EF534806	EF534848
*Geckoella collagensis*	CES 1136	Mumbai, Maharashtra	-	-	KC735087	KC735092
*Gekko gecko*	No ID	unknown	-	-	EF534813	EF534854
*Goniurosaurus araneus*	JFBM 15830	Vietnam	-	-	HQ 426286	HQ 426197
*Gymnodactlus amarali*	CHUNB 38646	Cocalzinho, Goiás, Brazil	-	-	HQ 426288	HQ 426199
*Heloderma suspectum*	TG 00068	Arizona, USA	-	-	AY662606	HQ426254
*Hemidactylus anamallensis 1*	CES 08029	Vadiyoor, Eravikulam, Tamil Nadu, India	KC735113	HM595680	HM622353	HM622368
*Hemidactylus anamallensis 2*	CES 08030	Vadiyoor, Eravikulam, Tamil Nadu, India	KC735114	KC735101	KC735088	KC735093
*Hemidactylus anamallensis 3*	CES 10002	Wayanad, Tamil Nadu, India	KC735115	KC735102	KC735089	KC735094
*Hemidactylus anamallensis 4*	CES 10003	Wayanad, Tamil Nadu, India	KC735116	KC735103	-	-
*Hemidactylus anamallensis 5*	CES 10004	Wayanad, Tamil Nadu, India	KC735117	KC735104	KC735090	KC735095
*Hemidactylus angulatus*	MVZ 245438	Nigeria, Togo Hills, Nkwanta	HQ426540	-	EU268306	EU268336
*Hemidactylus angulatus 1*	E1708.15	Kajiado District, Rift valley, Kenya	-	DQ120412	-	-
*Hemidactylus bowringii*	CES 08008	Sikkim, India	-	-	HM622354	HM622369
*Hemidactylus brookii 2*	CES 06080	Palakkad, Kerala, India	KC735118	HM595685	HM622355	HM622370
*Hemidactylus fasciatus 2*	-	Rabi, Gabon	-	-	EU268309	EU268339
*Hemidactylus frenatus 2*	CES 07035	Athirapalli, Valparai, Tamil Nadu, India	KC735119	KC735105	HM622371	HM622356
*Hemidactylus giganteus*	CES 07013	Nandi Hills, near Bangalore, Karnataka, India	KC735120	KC735106	-	-
*Hemidactylus giganteus*	CES 08013	Hampi, Karnataka, India	-	-	HM622357	HM622372
*Hemidactylus graniticolous*	CES 08028	Nilgiri Hills, Tamil Nadu, India	-	-	HM622361	HM622375
*Hemidactylus greefii*	CAS 219044	Praia da Mutamba, São Tome Island, São Tome and Principe	HQ426542	-	EU268308	EU268338
*Hemidactylus greefii*	E7014.4	Principe, Sao Tome and Principe	-	DQ120414	-	-
*Hemidactylus haitianus*	AMB 4189	Dominican Republic (1), Santo Domingo	HQ426543	-	-	-
*Hemidactylus haitianus 1*	HhaitiS	Matanzas, Matanzas province, Cuba	-	DQ120388	-	-
*Hemidactylus haitianus 2*	CAS 198442	near Santo Domingo, Nacional Dist., Dominican Republic	-	-	EU268311	EU268341
*Hemidactylus mabouia*	E609.20	Lake Nabugabo, Masaka District, Uganda	**-**	DQ120377	-	-
*Hemidactylus mabouia*	MCZ R-184446	Limpopo Province, South Africa	-	-	EU268300	EU268330
*Hemidactylus mabouia*	JME 1864	Wundanyi, Kenya	HQ426546	-	-	-
*Hemidactylus maculatus*	BNHS 1516	Zirad, Raigadh dist., Maharashtra, India	-	-	HM559707	HM559674
*Hemidactylus palaichthus*	LSUMZ H-12421	Roraima State, Brazil	-	-	EU268307	EU268337
*Hemidactylus persicus 2*	CES 08027	Nabh Dongar, Jaisalmer, Rajasthan, India	KC735121	KC735107	HM622362	HM622376
*Hemidactylus platyurus 2*	CES 08025	Kalimpong, West Bengal, India	-	-	HM622363	HM622377
*Hemidactylus robustus*	MVZ 248437	40 km South of Mipur Sakro, Thatta District, Pakistan	-	-	EU268315	EU268345
*Hemidactylus turcicus*	LSUMZ H-1981	Baton Rouge, Louisiana, USA	-	-	EU268299	EU268329
*Homonota fasciata*	TG 00085	Paraguay	-	-	EU 293629	EU 293697
*Lepidodactylus lugubris*	AMB 4111	Kirimati, Kiribati	-	-	EF534812	EF534853
*Lialis burtonis*	TG 00078	Provinsi Papua, Indonesia	-	-	EF 534782	EF 534822
*Narudasia festiva*	AMB 3243	Narudas, Namibia	-	-	EF534808	EF534850
*Nephrurus milii*	AMB 499	Western Australia, Australia	-	-	EF534780	EF534820
*Oedura marmorata*	AMS 143861	Queensland, Australia	-	-	EF 534779	EF 534819
*Paradelma orientalis*	QM-J56089	20 km N Capella, Queensland, Australia	-	-	HQ 426304	HQ 426215
*Phelsuma madagascariensis*	FG/MV 2002.797	Manongarivo, Madagascar	-	-	EF534811	AB081507
*Phyllodactylus xantii*	ROM 38490	Baja California Sur, Mexico	-	-	EF 534807	EF 534849
*Phyllodactylus xantii*	ROM 38490	*Baja California Sur, Mexico*	-	-	EF534807	EF534849
*Pristurus carteri*	TG 00083	*Yemen*	-	-	EF534803	EF534845
*Pygopus nigriceps*	AMB 53	Northern Territory, Australia	-	-	EF 534783	EF 534823
*Rhoptropus boultoni*	CAS 214713	*Twyfelfontein, Namibia*	-	-	EF534810	EF534852
*Sphaerodactylus elegans*	YPM 14795	Florida, USA	-	-	EF534787	EF534828
*Tarentola Americana*	MVZ 241223	13 km E of Pilon, Granma Province, Cuba	-	-	HQ 426332	HQ 426243
*Teratoscincus roborowskii*	TG 00070	*China*	-	-	EF534799	EF534841
*Thecadactylus solimoensis*	KU 214929	Cuzco Amazonico, Madre de Dios, Peru	-	-	EU 293644	EU 293711

Sequences generated by the authors have accession numbers starting with KC. For a complete list of C-*mos* and *12S rRNA* sequences see Feng et al. [25].

### Clarifying the position of *H. anamallensis* within the clade consisting of *Hemidactylus* and other closely related genera (*RAG-1* and *PDC* dataset)

**Figure 2:**
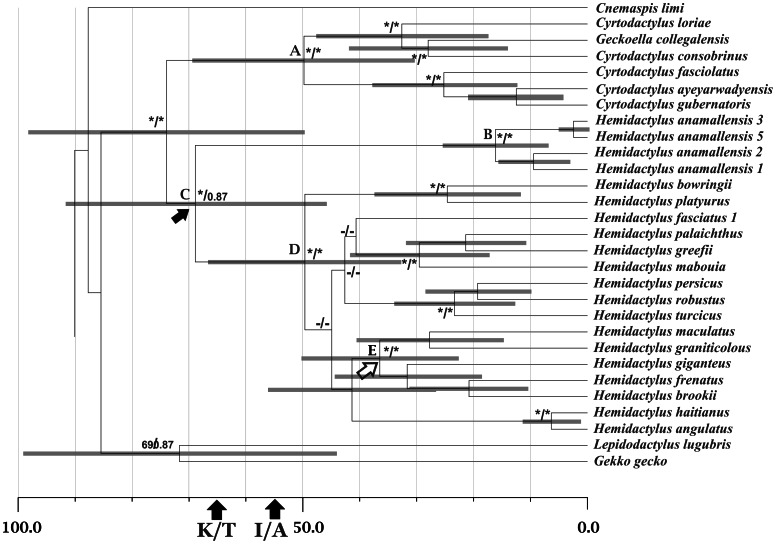
Bayesian estimates of dates based on *RAG-1* and *PDC* dataset. Bayesian posterior probabilities and maximum likelihood bootstrap supports are shown at the base of the nodes. Grey bars indicate the credible intervals. Black circle on the node represents the fixed date node and the hollow circle represents minimum age constraint node. Black arrow represents the node at which *H. anamallensis* split from the *Hemidactylus* lineage (68.9 mya) and white arrow represents the node at which Indian *Hemidactylus* lineage started radiating (36.47 mya). */* indicates the bootstrap support ≥90% and Bayesian posterior probability of 1, −/− indicates the bootstrap support ≤50% and Bayesian posterior probability of <0.5. K-T indicates Cretaceous-Tertiary boundary and I/A indicates the date of collision of India with Asian plate. For the complete tree see figure S1 and table S4.

In all the methods of phylogenetic inference, *H. anamallensis* emerged as sister to *Hemidactylus* and was separated from *Hemidactylus* by a long branch (Bayesian tree shown in figure 2). Genera *Cyrtodactylus* and *Geckoella* were sister to *Hemidactylus*-*H. anamallensis* clade. The overall topology of the Bayesian, ML and MP trees were similar with respect to the relationships among *Cyrtodactylus, Geckoella, Hemidactylus* and *H. anamallensis*. For a list of sequences used and their accession numbers see table 1.

### Divergence dates estimates

Bayesian estimation of divergence dates suggests that the ancestral lineage leading to *H. anamallensis* and the remaining *Hemidactylus* (node C) diverged from each other around 68.9 million years ago (mya) (95% HPD 45.15–92.65 mya) (figure 2, table 2). Additionally the lineage leading to the remaining *Hemidactylus* underwent radiation much later around 49.62 mya (Node D, 95% HPD 32.12–67.12 mya) (figure 2, table 2). The divergence dates estimated at the other nodes in this analysis were concordant with the divergence dates from previous studies [17]–[19].

**Table 2:** Estimated ages in Myr and in the corresponding 95% HPD for the nodes labelled in fig. 2.

Node	Age	95% HPD
A	49.79	30.19–69.39
B	16.12	7.6–24.64
C	68.9	45.15–92.65
D	49.62	32.12–67.12
E	36.47	19.89–53.05

The ages were obtained using uncorrelated lognormal clock in Bayesian estimation method BEAST.

## Discussion

The molecular data presented in the current study provided interesting insights into the phylogenetic position of *H. anamallensis* within Gekkonidae. The *C-mos* and *12S rRNA* dataset suggested that *H. anamallensis* was part of a large clade consisting of genera such as *Agamura*, *Cyrtodactylus*, *Cyrtopodian*, *Geckoella*, *Hemidactylus*, *Stenodactylus*, and *Tropiocolotes* (figure 1). This clade received high posterior probability and bootstrap support and, more importantly the members of this clade shared a 21 bp deletion that was not seen in any other gekkonid. Within the deletion clade *H. anamallensis* was sister to *Hemidactylus*. *H. anamallensis* and *Hemidactylus* were also retrieved as sister to each other by *RAG-1* and *PDC* dataset. Thus the nuclear markers support Bauer and Russell's [8] hypothesis that *H. anamallensis* might be a primitive *Hemidactylus*.

Interestingly in the *C-mos* gene, a 9 bp insertion was observed among *Hemidactylus* (figure 1b). This insertion was unique to the *Hemidactylus* lineage and was not shared with any other Gekkonid including *H. anamallensis*. Furthermore in the *RAG-1* + *PDC* tree *H. anamallensis* was separated form the rest of the *Hemidactylus* by a long branch. Thus among nuclear markers *H. anamallensis* appeared distinct from the remaining *Hemidactylus*.

Our divergence date estimates based on both fossils as well as biogeographical events suggested that the divergence between the lineage leading to *H. anamallensis* and the rest of the *Hemidactylus* lineage occurred around 68.9 mya (95% HPD 48.15–89.65) (figure 2, table 2) in the late Cretaceous. However, the remaining members of the *Hemidactylus* lineage radiated much later around 49.62 mya (95% HPD 36.12–63.12) (figure 2, table 2) in the Eocene. During the late Cretaceous period peninsular Indian landmass was isolated from all other landmasses having separated from Madagascar around 80 mya. Nevertheless fossil evidence suggested that peninsular India, during its northward journey, remained close to Africa and Eurasia until it collided with the Asian plate around 55 mya [20], [21]. Thus faunal links between peninsular Indian and these landmasses were maintained by vagile animals, which were able to surmount minor marine barriers [20]. Interestingly members of the deletion clade (figure 1a), which consisted of genera closely related to *H. anamallensis*, are distributed predominantly in Northern Africa and Asia. This distribution pattern suggested that basal radiation within this clade might have occurred on these landmasses. Furthermore during the early stages of this radiation one of the lineages might have dispersed on to the drifting peninsular Indian plate where it eventually evolved into *H. anamallensis*. Much later, around 49.62 mya, the genus *Hemidactylus* underwent radiation (figure 2, table 2) probably on the Asian plate [12] and dispersed to other parts of the world including peninsular India. Recent molecular studies on *Hemidactylus* revealed that India harboured an endemic radiation [14], [15]. According to our dating estimate, this Indian radiation occurred around 36.47 mya (Node E) (95% HPD 19.89–53.05 mya) (figure 2, table 2). Taken together these dates suggested that *Hemidactylus* arrived on the Indian plate after peninsular India collided with Asia. During this time *H. anamallensis* was already present in India, having dispersed on to drifting peninsular India before collision. In a recent molecular study a similar late Cretaceous dispersal of frogs on to drifting peninsular India has been reported [22].

Thus, the dating estimates suggests that *H. anamallensis* has a unique biogeographical history that appears to be very different from that of the remaining *Hemidactylus*. Additionally *H. anamallensis* also appears to be genetically distinct from the remaining *Hemidactylus*. Taken together, these results support the reassignment of *H. anamallensis* to a separate genus by resurrection of *Dravidogecko*, the genus to which *H. anamallensis* was previously assigned. In the past, authors have sunk *Dravidogecko* into *Hemidactylus*, as there were no morphological features that were unique to *Dravidogecko* [7], [8], [23]. According to Bauer et al. [8] the characteristic undivided lamellae seen in *H. anamallensis* is not unique to this species as it is shared with a highly derived lineage of ground dwelling *Hemidactylus* spp. of South Asia. They suggested that *H. anamallensis* was part of this highly derived lineage within the *H. brookii* complex. However the present study does not support this relationship as in both the phylogenies *H. anamallensis* is not sister to *H. brookii* within the *Hemidactylus* radiation. Thus this character (undivided lamellae) appears to have been secondarily derived in one of the lineages of *Hemidactylus*.

## Materials and Methods

### Sample collection and DNA sequencing

Genera that are purported to be closely related to *Hemidactylus* such as *Cyrtodactylus*, *Cyrtopodian*, *Geckoella* as well as *H. anamallensis* were collected opportunistically from across India (table 1). Total DNA was extracted from the tail clippings stored in absolute alcohol following standard proteinase K protocol [24]. Three nuclear, C*-mos*, recombination activation gene (*RAG-1*) and phosducin (*PDC*), and one mitochondrial marker, 12S ribosomal RNA (*12S rRNA*), were PCR amplified from the above samples. All PCR amplifications were carried out in 25 µl reaction volume, with 1.5 unit of Taq DNA polymerase (Bangalore Genei, Bangalore, India), 0.25 mM of dNTP's (Bangalore Genei), 2.0 mM of MgCl_2_, 1 ul of 0.5 mg/ml of BSA, 0.1 µM (Sigma) of each primer and 40 ng of DNA. Primer combinations and thermocycler conditions are given in supporting information (tables S1 & S2). PCR products were purified using QIAquick PCR Purification kit (Qiagen) and sequences were obtained commercially from Eurofins Biotech Pvt. Ltd. (Bangalore, India). For the remaining genera of the family Gekkonidae, sequences were downloaded from GenBank (table 1). Percent sequence generated for this study: C*-mos* 30%, *12S rRNA* 20%, *RAG-1*8%, *PDC* 8%.

### Phylogenetic analyses

The sequences generated here were combined with published sequences to derive two different datasets. First, to determine the phylogenetic position of *H. anamallensis* within Gekkonidae, the sequences generated by us were added to a combined dataset of the nuclear *C-mos* and mitochondrial *12S rRNA* genes generated by Feng et al. [25]. To clarify the position *H. anamallensis* within the clade consisting of *Hemidactylus* and other closely related genera: *RAG-1* and *PDC* datasets generated by Bauer et al. [13], Gamble et al. [17] and Bansal and Karanth [15] were used. In both the above datasets representatives from all the five clades of the *Hemidactylus* radiation were included. These sequences were aligned using ClustalW 1.6 [26] in the software MEGA v. 4.1 [27], using default parameters. These two datasets were then subjected to maximum parsimony (MP), maximum likelihood (ML) and Bayesian analyses. The two datasets could not be combined because there was a lack of overlap in sequence data between them. The *C-mos*+*12S rRNA* dataset generated by Feng et al. [25] had sequences largely for family Gekkonidae, thus this dataset was useful in inferring the position of *H. anamalensis* within Gekkonidae radiation. However *RAG-1 + PDC* dataset generated by Gamble et al. [17] had representatives of all the closely related families of Gekkonidae and therefore was useful in molecular dating (see below). Furthermore, in the case of *RAG-1+ PDC* extensive sequence data was available for *Hemidactylus* from previous works by Bauer et al. [13], and Bansal and Karanth [15]. Thus this dataset was also useful in clarifying the position of *H. anamalensis* within the clade consisting of *Hemidactylus* and other closely related genera.

The MP tree was derived through a heuristic search in in PAUP* version 4.0b10 [28] with tree bisection–reconnection branch swapping and 10 replicates of random addition options. Here transversions were weighted based on empirically determined transition/transversion ratios. Supports for various nodes were evaluated through 1000 replicates of bootstrapping in parsimony analysis. Phylogenetic inference using ML algorithm was also performed in PAUP with the substitution model chosen by MODELTEST [29] and tree bisection–reconnection branch swapping and 10 replicates of random addition options. Since PAUP does not allow for partitioning the dataset for ML search, another ML tree was derived in RAxML [30] wherein the dataset was partitioned. Bayesian analysis was run in Mr. Bayes version 3.1 [31] using the mixed model (see supporting information for partitioning scheme) with variable priors for 10^7^ generations with four chains, wherein sampling was undertaken for every 100 generations. All sample points before the stage when the Markov chain reached a stable likelihood value were discarded as burn-in determined in Tracer v 1.4.1 [32]. The remaining trees were imported into PAUP* to generate a majority-rule consensus tree and to derive posterior probabilities for each node. Gaps were treated as missing data for all analyses.

### Analysis of insertions and deletions (indels) in *C-mos* gene

*C-mos* is a proto-oncogene that encodes the protein serine/threonine kinase that regulates meiotic maturation in germ cells [33]. It is a single-copy gene that lacks introns and repetitive elements. Insertions and deletions in *C-mos* have been reported to be uncommon [34]. However, Han et al. [35] reported a 21 bp deletion in *C-mos* that was shared by some gekkonids. Additionally, our preliminary analysis suggested that members of the *Hemidactylus* radiation shared a 9 bp insertion. Given that indels are quite rare in coding regions, such changes could be used as phylogenetically informative characters for determine the position of *H. anamallensis*. Thus we checked the *C-mos* alignment for the presence of these indels in *Hemidactylus* (including *H. anamallensis*) and other related genera.

### Molecular dating

The *RAG-1* and *PDC* dataset (1439 characters) was also used to determine the divergence dates among *H. anamallensis*, *Hemidactylus* and other closely related genera. Independent calibrations from previously published studies [17]–[19] were used to constrain nodes in the divergence date analyses. Two out of five calibrations used in the previous studies were excluded from further analysis by the fossil cross- validation method used by Gamble et al. [17]. The excluded calibrations were (i) the minimum age of *Paradelma orientalis*/*Pygopus nigriceps* split, using the fossil *Pygopus hortulanus*, (ii) the maximum age of squamates, using the oldest known squamate fossil. The calibration points included and used to infer the divergence dates were: (i) Fossil *Primaderma nessovi* [36] was used to constrain the Helodermatidae/Anguidae split (exponential distribution, mean 3.0, offset 99.0). (ii) Two amber preserved specimens of *Sphaerodactylus spp.* [37], [38] were used to constrain the node constituting *Sphaerodactylus* species (exponential distribution, mean 5.0, offset 23.0). (iii) The split of *Teratoscincus scincus- Teratoscincus roborowskii* [39] which was purported to have occurred due to Tein Shan-Pamir uplift in western China, 10 Ma [40], [41] (Normal distribution, mean 10.0, SD 0.5)

The dataset was partitioned into two genes (*RAG-1* 1044 bp, *PDC* 395 bp) and the model of sequence evolution as mentioned in supporting information (table S3) was applied to both the partitions. Given that a strict clock model of molecular evolution is purported to be biologically unrealistic [42] a relaxed molecular clock model with uncorrelated lognormal distribution and Yule process tree prior (as recommended for species level phylogenies) were used. These analyses were undertaken in the program BEAST v 1.6.1 [43]. Base frequencies were estimated in BEAST, and gamma distribution categories were set to four. A default setting for substitution rate was used. The program was run for 5×10^7^ generations. Tracer v 1.4.1 [32] was used to determine convergence and effective sample sizes for the run.

## Supporting Information

Figure S1 **Bayesian estimates of dates based on** ***RAG-1*** **and** ***PDC*** **dataset.** Bootstrap supports and Bayesian posterior probabilities are shown at the base of the nodes. Grey bars indicate the credible intervals. K-T indicates Cretaceous-Tertiary boundary and I/A indicates the date of collision of India with Asian plate.
(TIF)

Table S1 **List of Primers used.**
(DOC)

Table S2 **Thermo cycler profile used for amplification of genes.**
(DOCX)

Table S3 **Partitioning scheme and model of sequence evolution for the genes in the datasets.** The datasets were partitioned according to the genes in both MrBayes and RAxML.
(DOCX)

Table S4 **Estimated ages (in Myr) of the nodes and the corresponding 95% CI for the nodes labelled in figure S1.**
(DOCX)
