# Supplementary figures and images for: Phylogenetic Analysis and Molecular Dating Suggest That Hemidactylus anamallensis Is Not a Member of the Hemidactylus Radiation and Has an Ancient Late Cretaceous Origin
Source: PLoS One. 2013 May 16;8(5):e60615. doi: 10.1371/journal.pone.0060615 (PMC3655972; doi:10.1371/journal.pone.0060615)

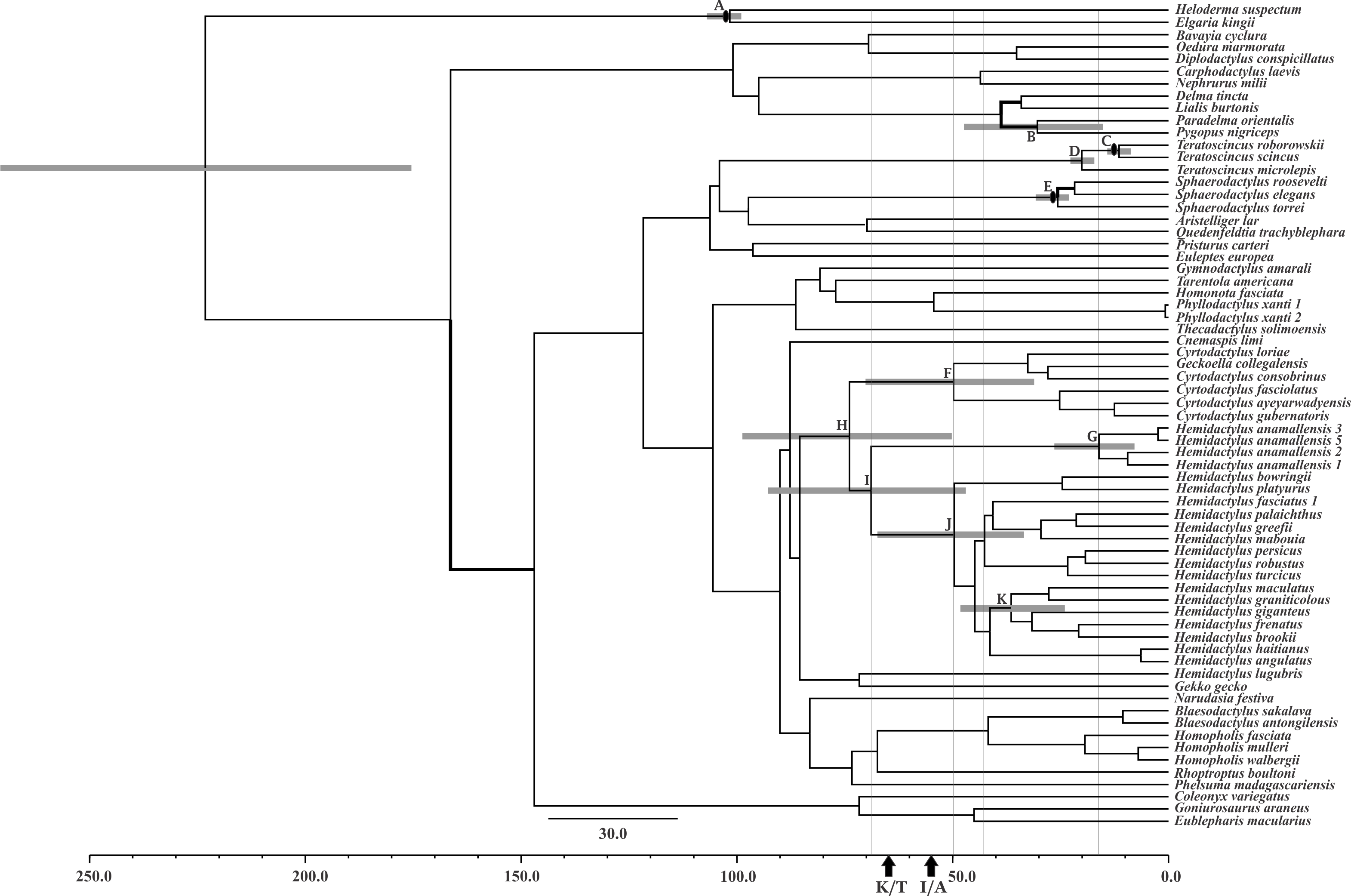

Supplement: Figure S1 — Bayesian estimates of dates based on RAG-1 and PDC dataset. Bootstrap supports and Bayesian posterior probabilities are shown at the base of the nodes. Grey bars indicate the credible intervals. K-T indicates Cretaceous-Tertiary boundary and I/A indicates the date of collision of India with Asian plate. (TIF) [file pone.0060615.s001.tif]
