# Supplementary material for: Phylogenetic Analysis and Molecular Dating Suggest That Hemidactylus anamallensis Is Not a Member of the Hemidactylus Radiation and Has an Ancient Late Cretaceous Origin
Source: PLoS One. 2013 May 16;8(5):e60615. doi: 10.1371/journal.pone.0060615 (PMC3655972; doi:10.1371/journal.pone.0060615)
Supplement: Table S1 — List of Primers used. (DOC) [file pone.0060615.s002.doc]

**Table S1:** List of Primers used.

| **Primer** | **Sequence** | **Reference** |
| --- | --- | --- |
| **12S ribosomal RNA (12S)** | | |
| 12S L1201 | 5'- AAA CTG GGA TTA GAT ACC CCA CTA T- 3' | Bansal and Karanth [15] |
| 12S H1486 | 5'-GAG GGT GAC GGG CGG TGT GT-3' | Bansal and Karanth [15] |
| **Recombination activating gene-1 (RAG-1)** | | |
| RAG1 F700 | 5’-GGAGACATGGACACAATCCATCCTAC-3’ | Bansal and Karanth [15] |
| R18 | 5’-GATGCTGCCTCGGTCGGCCACCTTT-3’ | Bansal and Karanth [15] |
| R13 | 5’- TCTGAATGGAAATTCAAGCTGTT-3’ | Bansal and Karanth [15] |
| RAG1 R700 | 5’-TTTGTACTGAGATGGATCTTTTTGCA-3’ | Bansal and Karanth [15] |
| **Phosducin (PDC)** | | |
| PHOF2 | 5’-AGATGAGCATGCAGGAGTATGA-3’ | Bansal and Karanth [15] |
| PHOR1 | 5’-TCCACATCCACAGCAAAAAACTCCT-3’ | Bansal and Karanth [15] |
| **C-*mos*** | | |
| G 73 | 5’-GCGGTAAAGCAGGTGAAGAAA- 3’ | Saint et al. [34] |
| G 74 | 5’-TGAGCATCCAAAGTCTCCAATC- 3’ | Saint et al. [34] |
