# Supplementary material for: Phylogenetic Analysis and Molecular Dating Suggest That Hemidactylus anamallensis Is Not a Member of the Hemidactylus Radiation and Has an Ancient Late Cretaceous Origin
Source: PLoS One. 2013 May 16;8(5):e60615. doi: 10.1371/journal.pone.0060615 (PMC3655972; doi:10.1371/journal.pone.0060615)
Supplement: Table S2 — Thermo cycler profile used for amplification of genes. (DOCX) [file pone.0060615.s003.docx]

**Table S2:** Thermo cycler profile used for amplification of genes.

| **PCR Product** | **Length** | **PCR Profile** |
| --- | --- | --- |
| **Mitochondrial Genes** | | |
| 12S | 399 bp | 94°C: 5 minutes, (94°C: 30 seconds, 48°C: 45 seconds, 72°C: 1 minute) x 35 cyles, 72°C: 5 minutes, Hold at 4°C |
| **Nuclear Genes** | | |
| RAG-1 | 650 bp | (94°C: 3 minutes, 48°C or 55°C: 45 seconds, 72°C: 1 minute),(94°C: 30 seconds, 48°C or 55°C: 45 seconds, 72°C: 1 minute) x 35 cyles, 72°C: 6 minutes, Hold at 4°C. |
| PDC | 450 bp | 95°C: 12 minutes, (94°C: 1 minute, 56°C: 1 minute, 72°C: 1 minute) x 35 cyles, 72°C: 5 minutes, Hold at 4°C. |
| C-*mos* | 500 bp | (94°C: 3 minutes, 48°C or 55°C: 45 seconds, 72°C: 1 minute),(94°C: 30 seconds, 48°C or 55°C: 45 seconds, 72°C: 1 minute) x 35 cyles, 72°C: 6 minutes, Hold at 4°C. |
