# Supplementary material for: Phylogenetic Analysis and Molecular Dating Suggest That Hemidactylus anamallensis Is Not a Member of the Hemidactylus Radiation and Has an Ancient Late Cretaceous Origin
Source: PLoS One. 2013 May 16;8(5):e60615. doi: 10.1371/journal.pone.0060615 (PMC3655972; doi:10.1371/journal.pone.0060615)
Supplement: Table S3 — Partitioning scheme and model of sequence evolution for the genes in the datasets. The datasets were partitioned according to the genes in both MrBayes and RAxML. (DOCX) [file pone.0060615.s004.docx]

**Table S3:** Partitioning scheme and model of sequence evolution for the genes in the datasets. The datasets were partitioned according to the genes in both MrBayes and RAxML.

| **Model of sequence evolution** | **Parameters** |
| --- | --- |
| **C-*mos* :** TrN+G | Nst= 6, rates=G, K= 6  Base frequencies:  freqA = 0.3102  freqC = 0.2097  freqG = 0.2117  freqT = 0.2684 |
| **12S rRNA :** GTR+I+G | Nst= 6, rates=G+I, K= 10  Base frequencies:  freqA =0.2981  freqC = 0.2385  freqG = 0.2191  freqT = 0.2442 |
| **RAG-1:** TIM+G | Nst= 6, rates=G, K= 7  Base frequencies:  freqA = 0.3297  freqC = 0.2253  freqG = 0.2149  freqT = 0.2301 |
| **PDC:** TrN+I+G | Nst= 6, rates= G+I, K= 7  Base frequencies:  freqA = 0.2982  freqC = 0.2339  freqG = 0.2555  freqT = 0.2124 |
