# Supplementary material for: Phylogenetic Analysis and Molecular Dating Suggest That Hemidactylus anamallensis Is Not a Member of the Hemidactylus Radiation and Has an Ancient Late Cretaceous Origin
Source: PLoS One. 2013 May 16;8(5):e60615. doi: 10.1371/journal.pone.0060615 (PMC3655972; doi:10.1371/journal.pone.0060615)
Supplement: Table S4 — Estimated ages (in Myr) of the nodes and the corresponding 95% CI for the nodes labelled in figure S1. (DOCX) [file pone.0060615.s005.docx]

**Table S4:** Estimated ages (in Myr) of the nodes and the corresponding 95% CI for the nodes labelled in figure S1.

| NODE | DATE | CI |
| --- | --- | --- |
| A | 101.66 | 4.7 |
| B | 30.36 | 14.62 |
| C | 11.01 | 2.0 |
| D | 20.03 | 2.3 |
| E | 25.68 | 3.78 |
| F | 49.79 | 19.6 |
| G | 16.12 | 8.52 |
| H | 73.91 | 24.2 |
| I | 68.9 | 23.75 |
| J | 49.62 | 17.5 |
| K | 36.47 | 16.58 |
|  |  |  |
